# Defining symptoms of malaria in India in an era of asymptomatic infections

**DOI:** 10.1186/s12936-020-03310-9

**Published:** 2020-07-06

**Authors:** Anna Maria van Eijk, Asad S. Mannan, Steven A. Sullivan, Jane M. Carlton

**Affiliations:** Center for Genomics and Systems Biology, Department of Biology, New York University, New York, NY 10003 USA

**Keywords:** Malaria, Urban, Rural, Asymptomatic infection, Microscopy, PCR, Rapid diagnostic test

## Abstract

**Background:**

Malaria is a major public health problem in India. Data from surveys totaling 3031 participants at three sites revealed a high proportion of asymptomatic infections, complicating diagnosis. The aim of this study was to identify differences in complaints and symptoms between sites, and factors associated with asymptomatic *Plasmodium* infections.

**Methods:**

Published data from community-based cross-sectional studies conducted between 2012 and 2015 in Nadiad (Gujarat), Chennai (Tamil Nadu), and Rourkela (Odisha) as part of the Center for the Study of Complex Malaria in India were analysed. Complaints and symptoms were systematically recorded, and *Plasmodium* infections confirmed using microscopy, rapid diagnostic tests (RDTs), and polymerase chain reaction (PCR). Multivariate analyses were conducted to determine the association between general symptoms and age, season, or gender, and factors associated with asymptomatic *Plasmodium* infections were assessed.

**Results:**

Complaints of any illness were lowest in Chennai (17.7%), 30.6% in Rourkela and 42.7% in Nadiad. Complaints were more often reported for children; gender differences were noted in Rourkela only. In Nadiad, 7.0% of 796 participants were positive for malaria by PCR (32% *Plasmodium falciparum*); 78.6% had a history of fever or documented fever, 14.3% had other symptoms, and 7.1% were “truly asymptomatic”. For Chennai this was 29.2%, 4.2% and 66.7% respectively, with a malaria prevalence of 2.6% by PCR of 928 participants (29% *P. falciparum*). In Rourkela, with 7.7% of 1307 participants positive for malaria by PCR (82% *P. falciparum*), the percentages were 35.6%, 24.8% and 39.6%, respectively. In Rourkela, asymptomatic infections were associated with young age and male gender (microscopy or RDT), and with rainy season (PCR). In the same site, participants with *Plasmodium vivax* were more likely to be asymptomatic (11/18 or 61.1%) than persons with *P. falciparum* mono-infections (27/78 or 34.6%); gametocytes for *P. falciparum* were evenly distributed between symptomatic and asymptomatic infections (2/53 vs. 2/49, respectively). The addition of the symptoms “headache”, “aches” and “chills” to fever improved the case-definition of symptomatic malaria.

**Conclusion:**

There were considerable differences in complaints at the three sites in India. Malaria and asymptomatic infections differ by region, indicating that malaria elimination will require localized approaches.

## Background

Malaria is still a major cause of morbidity and mortality in India, although the numbers are decreasing. In 2018, the National Vector Borne Disease Control Programme (NVBDCP) estimated that approximately 500,000 people suffered from malaria (63% *Plasmodium falciparum*), and less than 100 persons died [1]. These numbers are likely an underestimate; the World Malaria Report estimated that only 16% of cases may be detected by the Indian malaria surveillance system [2]. Malaria in India is complex, with multiple *Plasmodium* species and *Anopheles* vectors, and a wide variety of endemic settings [3]. *Plasmodium vivax* can be detected throughout the country, whereas *P. falciparum* is more common in the Eastern and North-Eastern states. The major malaria vector is *Anopheles culicifacies*, estimated to be associated with 60–65% of the malaria disease burden in India [3]. *Anopheles stephensi* is present in urban and peri-urban areas, and has been associated with serious malaria outbreaks in South India [4], whereas *Anopheles fluviatilis* is present in the forests and foothills, and *Anopheles minimus* mainly in the North-Eastern states [3]. The diverse malaria settings make the goal of malaria elimination challenging; one size does not fit all, and multiple localized strategies are needed, a stance supported by the National Framework for Malaria Elimination in India [5].

In the past in the absence of a diagnostic test, it was common to diagnose malaria based only on symptoms. Several studies have evaluated if specific symptoms or combinations of symptoms were reliable to diagnose malaria [6–8]; however, the symptoms of malaria are not very specific and can be caused by many diseases. In addition, malaria can present in unusual ways or can be part of co-morbidities [9–11]. Microscopy used to be the other main means of diagnosing malaria. More recent methods include malaria rapid diagnostic tests (RDTs) and molecular methods, such as polymerase chain reaction (PCR). RDTs are now widely used by local NVBDCP clinics and by community health workers (ASHAs) in India. Malaria testing using PCR requires expensive equipment and reagents as well as trained staff, which limits its use in local clinics. The greater sensitivity of PCR for detecting *Plasmodium* parasites has resulted in the introduction of the terms ‘submicroscopic’ and ‘subpatent’ malaria, referring to cases where the number of malaria parasites is below the detection level of microscopy or RDT [12, 13]. Surveys have repeatedly shown that not all cases of malaria present with symptoms; this phenomenon was well-known in African countries in areas of high malaria transmission intensity but is now also documented in areas with low malaria transmission and for both species of malaria parasites common in India [14]. Asymptomatic infections are important as a hidden reservoir of *Plasmodium* parasites [14], and can contribute to continued transmission. In addition, asymptomatic infections can still have subtle adverse effects on health [15, 16].

Symptoms of malaria among the general population can differ by region, depending on prevailing diseases [17]. A published study from the National Institutes of Health-funded International Center of Excellence for Malaria Research describing cross-sectional malaria surveys conducted at three sites in India included a detailed questionnaire on symptoms [3, 18]. Here this questionnaire data was used to assess the distribution of symptoms by region, age, and gender, and the association of symptoms with malaria. The goal was to determine the proportion of malaria associated with fever (documented fever or a history of fever in the past 48 h), or other symptoms (without fever), and the proportion of truly asymptomatic infections in these different regions.

## Methods

### Study sites and population

The three study sites have been described previously [18, 19]. Briefly, Chennai, the coastal capital city of the southern state of Tamil Nadu, has rainfall from October to December as part of the northeast monsoon, and again during the southwest monsoon between July and August [20]. Malaria transmission is predominantly *P. vivax*, and is perennial, peaking between July and October. Participants were enrolled from Besant Nagar, an urban area adjacent to the ocean. Nadiad town, in the central Kheda district of the western coastal state of Gujarat, receives the majority of its rain during the southwest monsoon season (June–September) [20]. Malaria there is unstable (hypo-endemic), with *P. vivax* and *P. falciparum* prevalence rates oscillating throughout the year based on the rainfall. Participants were enrolled from the peri-urban and rural areas adjacent to Nadiad town. Rourkela city in the Sundargarh district of the eastern state of Odisha receives rains during the southwest monsoon season (June–September) and some rainfall during the retreating northeast monsoon (December–January) [20]. Malaria displays meso- to hyper-endemic transmission in Odisha, with *P. falciparum* as the major infecting species. Insecticide treated nets are distributed for free in malarious areas by the Government of Odisha. Subjects were enrolled from peri-urban and rural areas around Rourkela. *An. culicifacies* is the major vector in Nadiad, *An. stephensi* in Chennai, and *An. fluviatilis* in Rourkela [3].

### Study designs

A census was conducted before the start of the surveys as described previously [19]. Cross-sectional surveys were conducted in household samples from the census over 2 years. Briefly, persons aged 12 months to 69 years were eligible after obtaining consent; pregnant women or persons with severe anaemia (haemoglobin < 7 g/dl) were excluded. After consent, individuals underwent a physical examination, were asked questions about their malaria history and symptoms at the time of the interview and had finger-prick blood collected for haemoglobin measurement, blood smear, RDT and PCR. Persons with a positive RDT were treated as per the national guidelines: *P. vivax*: chloroquine 25 mg/kg over 3 days and primaquine 0.25 mg/kg for 14 days; *P. falciparum* artesunate 4 mg/kg for 3 days in combination with sulfadoxine 25 mg/kg and pyrimethamine 1.25 mg/kg on the first day and primaquine 0.75 mg/kg [21]. Treatment was not directly observed.

### Diagnostic methods

Several diagnostic tests were used [18]. Haemoglobin level was assessed using HemoCue (Ängelholm, Sweden). Thin and thick smears were Giemsa-stained and examined by microscopy. Slides were read by two microscopists, and a third microscopist was used if there was disagreement. All samples, whether positive or negative by microscopy, underwent DNA extraction by QIAamp DNA blood Mini Kits (Qiagen Inc., Valencia, CA), and a modified nested, multiplex-PCR method targeting the 18S small subunit ribosomal protein (SSU rDNA) was used for species-specific detection of *Plasmodium* parasites [22, 23]. RDT was performed as per the manufacturer’s instruction.

### Data analysis

Data for the cross-sectional studies are available in the Clinical Epidemiology Database, ClinEpiD (https://clinepidb.org), under “Study: India ICEMR Cross-Sectional”. Data were exported into Stata (Stata/IC version 14.2, StataCorp LP, College Station, USA) for analysis. At all three study sites, the distribution of any symptoms by age, gender and season was examined first, followed by an evaluation of symptomatic and asymptomatic malaria. Malaria was defined in three groups: ‘Symptomatic and fever’ defined as *Plasmodium*-positive by PCR plus fever (documented fever, a body temperature of ≥ 37.5 °C or a history of fever in the past 48 h); ‘Symptomatic and other’ defined as *Plasmodium*-positive by PCR and other symptoms (not fever), and ‘Truly asymptomatic infection’. Parasite infections detected by PCR but not by microscopy were classified as ‘submicroscopic’ and infections detected by PCR but not by RDT as ‘subpatent’. For anaemia, an age- and gender-appropriate definition was used (haemoglobin < 11 g/dl if age < 5 years, < 11.5 g/dl if age ≥ 5 and < 12 years, < 12 g/dl if age > 12 and age < 15, < 12 g/dl if age ≥ 15 and female, and < 13 g/dl if ≥ 15 and male) [24]. Characteristics were weighted by age and gender using the household information obtained from the census (svy procedure in Stata 14.2); however, the analyses of symptoms versus malaria test results were not weighted because of our interest in the direct relationship between malaria and symptoms. The Fisher’s exact test was used for cross-tabulations, and the t-test for the comparison of haemoglobin across groups. Asymptomatic infections were examined for associations with age, gender, season, anaemia, a history of travel, or anti-malarial treatment in the past 2 weeks, or a history of malaria in the past year, use of repellents, and use of insecticide treated nets (in Rourkela only since nets were not used in Chennai or Nadiad). Generalized linear regression models with a log link and binomial distribution were used for multivariate analyses, and Poisson regression with a robust variance estimator was used for models which did not converge [25]; variables with a p-value ≥ 0.05 were removed from multivariate models, but age, gender and season were kept in the models to allow comparison across models. A p-value < 0.05 was considered significant.

## Results

### Characteristics of participants by region

Rourkela participants were younger and less educated than participants in the other sites and were more likely to have a history of malaria in the past year (Table 1). A recent history of travel was more common in Chennai and Nadiad. Recent anti-malarial use was 6% in Nadiad but significantly lower in the other sites. Insecticide-treated nets (ITNs) were only used in Rourkela, while the use of personal mosquito repellents was common in Chennai. Anaemia was particularly common in Nadiad (63%), and least common in Chennai (35%).

**Table 1:** Characteristics of survey participants in three sites in India, 2012–2014

	Cross-sectional surveys		
	Chennai % or mean (95% CI)	Nadiad % or mean (95% CI)	Rourkela % or mean (95% CI)
Number of participants	928	796	1307
Time period	Dec 12–Oct 14	May 13–Sept 14	Jan 13–Sept 14
Median age, interquartile range	29.0, 17.0–43.0	30.0, 17.0–45.0	25.0, 12.0–40.0
Age groups (%)			
< 5 years	6.3 (4.3–9.2)	5.4 (3.5–8.3)	8.8 (7.5–10.3)*
5–14 years	14.0 (11.7–16.7)	15.4 (13.0–18.1)	21.3 (19.3–23.5)
15+ years	79.7 (76.3–82.7)	79.2 (75.8–82.2)	69.9 (67.5–72.2)
Male	50.4 (46.7–54.0)	51.6 (48.0–55.2)	50.5 (47.8–53.2)
Among persons ≥ 18 years			
Highest level education			
None (%)	12.6 (10.3–15.4)	29.8 (26.3–33.5)	40.3 (37.0–43.6)^†^
Primary school (%)	25.7 (21.6–30.3)	49.6 (45.6–53.5)	52.8 (49.3–56.3)
Secondary or higher (%)	61.7 (57.3–65.9)	20.7 (17.7–24.1)	6.9 (5.4–8.8)
Salaried employment (%)	27.4 (24.2–30.9)	5.8 (4.1–8.0)	1.9 (1.2–3.0)^†^
History and symptoms			
History of malaria last year	5.9 (4.5–7.6)	4.6 (3.1–6.7)	25.9 (23.6–28.2)*
History of travel past 2 weeks	12.5 (10.3–15.1)	11.1 (9.0–13.7)	2.6 (1.9–3.6)*
Anti-malarial use past 2 weeks	0.3 (0.1–0.8)	5.7 (4.2–7.7)	1.6 (1.1–2.4)^†^
Use of nets	6.0 (4.3–8.3)	14.0 (11.5–17.0)	85.3 (83.5–87.0)^†^
Use of ITNs	0	0.6 (0.3–1.5)	28.4 (26.2–30.9)^†^
Use of repellents^a^	45.6 (42.0–49.4)	39.5 (36.1–43.1)	32.5 (29.8–35.3)^†^
Anaemia (%)^b^	35.4 (32.2–38.8)	62.5 (59.0–65.9)	48.8 (46.0–51.7)^†^

Weighted by age and gender. This table has been published before because the publication involved the same study population [18]

*CI* confidence interval, *ITN* insecticide treated net (long lasting insecticide treated net or a net treated within the last 6 months)

*p < 0.05 comparing Rourkela to Chennai and Nadiad

^†^p < 0.05 comparing to each other

^‡^p < 0.05 comparing Chennai to Nadiad and Rourkela

^§^p < 0.05 comparing Nadiad to Chennai and Rourkela

**p < 0.05 comparing Chennai to Nadiad

^a^Repellents include the use of coils, vaporizers, mats or creams for the prevention of mosquito annoyance

^b^Age and gender appropriate definition: Haemoglobin < 11 g/dl if age < 5 years, < 11.5 g/dl if age ≥ 5 and < 12 years, < 12 g/dl if age > 12 and age < 15, < 12 g/dl if age ≥ 15 and female, and < 13 g/dl if ≥ 15 and male

### Symptoms by region, age, gender and season

**Fig. 1:**
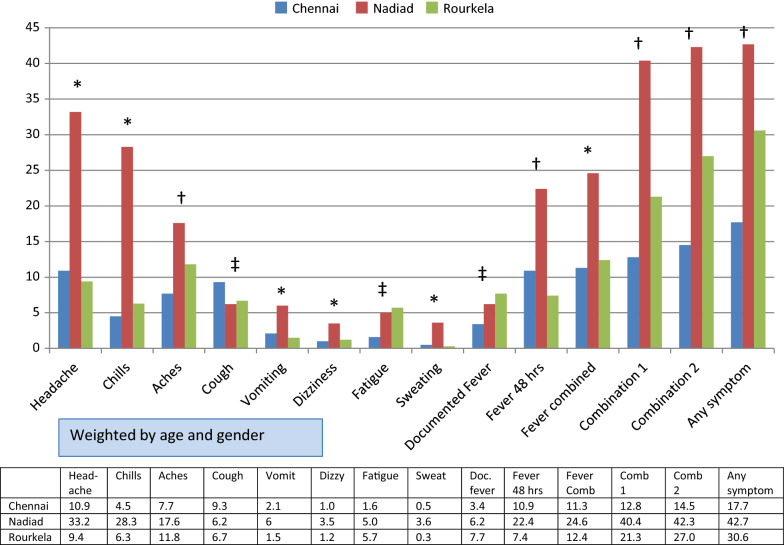
Symptoms as percentage in study population by region, India, 2013–2015. Definitions: Documented fever: Axillary temperature of ≥ 37.5 °C, Fever combined: documented fever or a history of fever in the past 48 h, Combination 1: headache or chills or aches, Combination 2: fever (fever combined) or headache or chills or aches. *p < 0.05 comparing Nadiad vs. other sites. ^†^p < 0.05 comparing sites with each other. ^‡^p < 0.05 comparing Chennai vs. other sites

There were considerable regional differences in general symptoms (Fig. 1: any symptoms 17.7% in Chennai, 42.7% in Nadiad, and 30.6% in Rourkela); many symptoms were more common in Nadiad (headache, chills, aches, vomiting, dizziness, fever or the combination of symptoms). In Chennai, younger age groups were more likely to have complaints of cough and vomiting, and to have documented fever, compared to older people (15+ years), but no differences by gender or season were detected (Additional file 1: Table S1A). In Nadiad, younger age groups were more likely to have chills, cough, dizziness, and fever compared to older age groups, and complaints of chills, aches, and cough were less likely to occur in the rainy season (Additional file 1: Table S1B). In Rourkela, aches were less likely to occur in the young age groups, but other complaints were more common in the young age group (chills, cough, and fever) compared to older people (Additional file 1: Table S1C). In contrast to Nadiad, complaints were more likely in the rainy season in Rourkela. Rourkela was also the only site with gender differences in complaints (chills more common among males, and headache and aches more common among females, Additional file 1: Table S1C).

### Asymptomatic infections

**Fig. 2:**
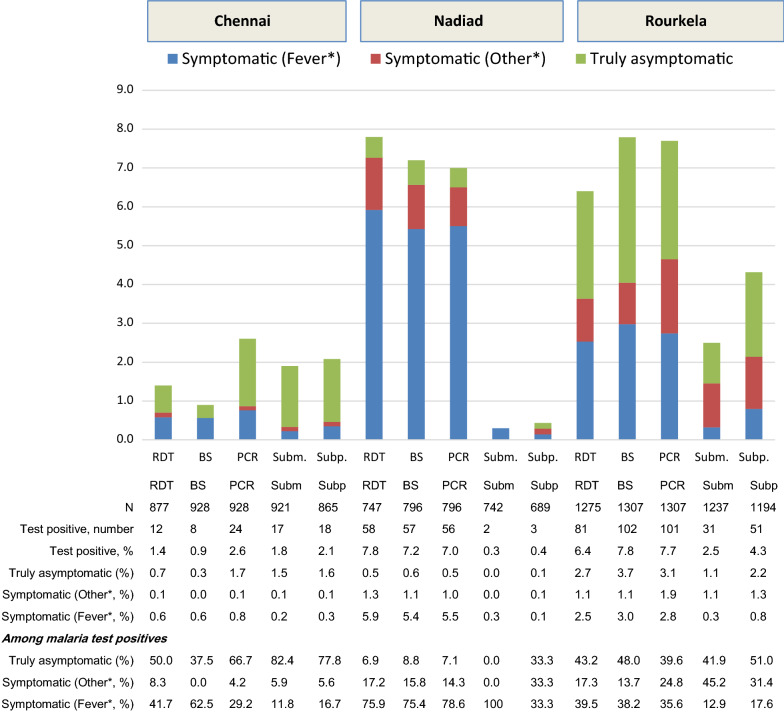
Symptomatic and asymptomatic malaria in three regions in India using documented fever or a history of fever in the past 48 h* as definition of symptomatic malaria, cross-sectional surveys, 2012–2015. *BS* blood smear (microscopy), *PCR* polymerase chain reaction, *RDT* rapid diagnostic test, *Subm* submicroscopic (detected by PCR but not by microscopy), *Subp* subpatent (detected by PCR but not by RDT). *Fever defined as a history of fever in the past 48 h or documented fever (a body temperature of 37.5 °C or more) at the time of visit. Symptomatic (other) indicates persons without fever as defined before but with other symptoms such as chills, headache, ache, cough, fatigue etc

The prevalence of malaria in Chennai as determined by all tests was low, ranging from 0.9% by microscopy to 2.6% by PCR (Fig. 2), with 29.2% of infections identified as *P. falciparum* by PCR. Malaria mainly presented with fever; presentations with symptoms other than fever, such as headaches and aches, were uncommon (range 0–8% among *Plasmodium* positive persons, depending on test). A total of 67% of infections detected by PCR and 82.3% of submicroscopic infections were truly asymptomatic. The proportion of truly asymptomatic infections among *Plasmodium* positive persons was higher when detected by PCR (66.7% of malaria detected) than by microscopy (37.5%) or RDT (50%, Fig. 2), and was higher among submicroscopic and subpatent infections (82.4% and 77.8%, respectively).

In Nadiad, the prevalence of malaria was 7-8% (32.1% *P. falciparum*, detected by PCR), with most malaria infections presenting with fever (range 75.4% for microscopy and 79% for PCR). Depending on the test, 14–17% presented with symptoms other than fever, with as most common symptoms headache, chills, and aches; 7–9% were truly asymptomatic (Fig. 2).

Although malaria in Rourkela (6–8%, 82.2% *P. falciparum* detected by PCR) was close to the prevalence in Nadiad, only a minority presented with fever (range 39.5% by RDT and 35.6% by PCR) or other symptoms (range 24.8% detected by PCR and 13.7% by microscopy); asymptomatic infections ranged from 39.6% detected by PCR to 51% among subpatent infections (Fig. 2). The most common symptoms in the absence of fever were headache, aches, chills and cough. Among persons with *P. falciparum*, parasite densities were significantly higher among persons with fever (geometric mean 3135 parasites/µl, 95% confidence interval [CI] 1459–6735, n = 35) compared to persons with asymptomatic malaria (geometric mean 765 parasites/µl, 95% CI 476–1231, n = 34, p = 0.002) and persons with symptoms other than fever (geometric mean 546 parasites/µl, 95% CI 161–1853, n = 12, p = 0.020). Few gametocytes of *P. falciparum* were detected (7 or 0.5%); among persons with infections they were evenly distributed between symptomatic and asymptomatic infections (2/53 vs. 2/49, respectively, p = 1.000), but gametocytes were more common among people with asexual parasitaemia compared to people without parasitaemia (4/102 vs. 3/1205, p = 0.001). Persons with *P. vivax* were more likely to be asymptomatic than persons with *P. falciparum* (microscopy: 12/14 vs. 35/82, respectively, p = 0.003; PCR: 11/18 for *P. vivax* and 27/78 for *P. falciparum*, p = 0.060). In the same site, associations were noted between asymptomatic infections and young age and male gender as detected by microscopy or RDT, and with rainy season as detected by PCR (Table 2). Although persons with asymptomatic infections had lower mean haemoglobin levels compared to persons without malaria detected by microscopy (asymptomatic malaria mean 11.5 g/dl, standard deviation [sd] 1.7 n = 47 vs. no malaria mean 12.0 g/dl, sd 1.6, n = 1101 by microscopy, p = 0.035), there was no effect on anaemia (25/47 or 53.2% vs. 558/1101 or 50.7%, p = 0.768, detected by microscopy; RDT results similar; no difference in haemoglobin by PCR).

**Table 2:** The association between age, gender and season and asymptomatic malaria in Rourkela (multivariate models), 2013–2015

	Asymptomatic malaria by RDT			Asymptomatic malaria by microscopy			Asymptomatic malaria by PCR		
	n/N (%)	Adjusted risk ratio, 95% CI	p	n/N (%)	Adjusted risk ratio, 95% CI	p	n/N (%)	Adjusted risk ratio, 95% CI	p
Age group									
0–4	5/116 (4.3)	2.55, 0.93–6.96	0.069	10/116 (8.6)	*2.77, 1.37–5.61*	0.005	4/116 (3.5)	1.12, 0.40–3.14	0.835
5–14	16/242 (6.6)	*4.00, 1.97–8.11*	< 0.001	13/239 (5.4)	1.78, 0.93–3.43	0.082	10/236 (4.2)	1.42, 0.69–2.90	0.338
15+ years	14/903 (1.6)	Reference		26/899 (2.9)	Reference		26/894 (2.9)	Reference	
Gender									
Male	22/565 (3.9)	1.84, 0.94–3.63	0.077	30/562 (5.3)	*1.79, 1.02–3.15*	0.044	24/551 (4.4)	1.81, 0.97–3.38	0.064
Female	13/696 (1.9)	Reference		19/692 (2.8)	Reference		16/695 (2.3)	Reference	
Season									
Rainy	14/544 (2.6)	0.94, 0.48–1.81	0.843	21/537 (3.9)	1.03, 0.60–1.80	0.905	24/526 (4.6)	*2.05, 1.10–3.82*	0.024
Dry	21/717 (2.9)	Reference		28/717 (3.9)	Reference		16/720 (2.2)	Reference	

Reference group: persons without malaria by this test. Asymptomatic malaria defined as malaria in the absence of any symptom (truly asymptomatic). Adjusted for age, gender and season. Adjusted risk ratios with a confidence interval > 1 are in italics

*CI* confidence interval, *RDT* rapid diagnostic malaria test, *PCR* polymerase chain reaction

Compared to the absence of malaria, asymptomatic infection was not associated with personal repellent use, travel, or anti-malarial treatment in the past 2 weeks at any site, or with ITN use in Rourkela, or with haemoglobin, anaemia, or *Plasmodium* species in Chennai or Nadiad; unfortunately, the information on parasite density in Chennai or Nadiad was too sparse to explore the association with asymptomatic infections. In Chennai, a history of malaria in the past year was more common among persons with asymptomatic infections than persons without malaria, as detected by RDT (2/51 vs. 4/820, p = 0.043), but not as detected by any other malaria test.

**Fig. 3:**
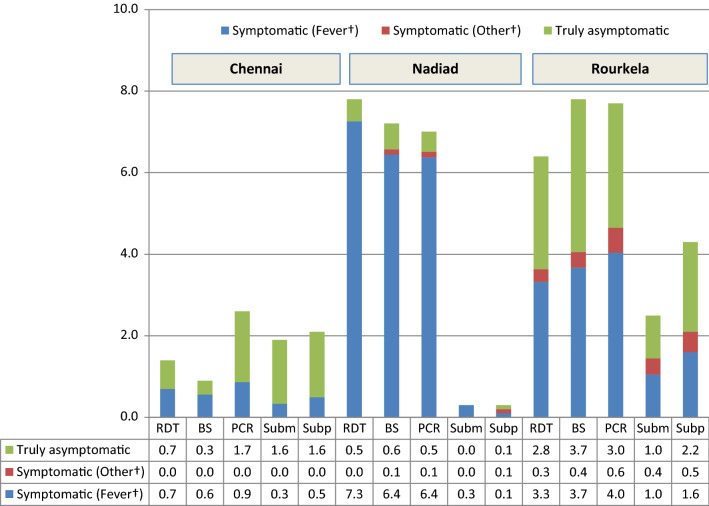
Symptomatic and asymptomatic malaria in three regions in India using documented fever or a history of fever in the past 48 h or presence of chills or aches or headache* as definition of symptomatic malaria, cross-sectional surveys, 2012–2015. *BS* blood smear (microscopy), *PCR* polymerase chain reaction, *RDT* rapid diagnostic test, *Subm* submicroscopic (detected by PCR but not by microscopy), *Subp* subpatent (detected by PCR but not by RDT). *Fever defined as a history of fever in the past 48 h or documented fever (a body temperature of 37.5 °C or more) at the time of visit, or chills or aches or headache. ^†^Symptomatic (other) indicates persons without fever as defined before or chills or headache or aches but with other symptoms such as fatigue etc

When considering any of the symptoms headache, aches, chills, or fever as a definition of symptomatic malaria, the number of malaria cases with symptoms other than these was reduced in Rourkela and Nadiad, and disappeared in Chennai, indicating that all patients in Chennai presented with one of these symptoms (Fig. 3). However, symptoms alone were insufficient to diagnose malaria. Using RDT and PCR as the gold standards of diagnosis, this combination of symptoms had a positive predictive value of just 4.8% and 5.2% in Chennai, 18.0 and 15.4% in Nadiad, and 11.7% and 14.5% in Rourkela.

## Discussion

India aims to eliminate malaria [5]. To do so it is important for all symptomatic and, if feasible, asymptomatic cases to be identified and treated. However, malaria in India is heterogeneous, and in addition, many diseases present with symptoms similar to it [11]. In the three regions of India with different malaria endemicities studied here, malaria prevalence was low (detected by PCR: 2.6%, 7.0% and 7.7% in Chennai, Nadiad and Rourkela, respectively). Fever was the most common symptom of malaria; chills, aches, and headache were common symptoms reported among persons without fever but with a positive malaria test. There were considerable regional differences in (truly) asymptomatic malaria: 7.1% of all positive malaria cases detected by PCR in Nadiad, 39.6% in Rourkela and 66.7% in Chennai. In Rourkela, asymptomatic infection was associated with young age and male gender as detected by microscopy, and with season as detected by PCR.

Different patterns of general symptoms were observed, with the lowest number of any symptoms in Chennai, and the highest number of any symptoms in Nadiad. Patterns of symptoms were higher in the younger age groups and were more common in the dry season in Nadiad, whereas in Rourkela they were more common in the rainy season. Only in Rourkela were gender differences observed for some symptoms (headaches and aches more common among women, chills more common among men). These patterns in symptoms by region indicates that the burden of disease in each region is different. It has been reported that cultural factors can contribute to differences in symptom experiences and health-seeking behavior [26]. Since several other diseases including influenza, dengue, and chikungunya have overlapping symptoms and all are prevalent in our study areas, it may be hard to differentiate between them [27].

It is not always apparent whether asymptomatic infections will develop into symptomatic malaria cases and be cleared by treatment with anti-malarial drugs or will be cleared by the person’s immune system without intervention. Submicroscopic asymptomatic parasite levels can persist after treatment, if parasites are drug resistant or if inadequate doses of anti-malarials have been used. An association with recent treatment was not detected in this analysis, but in Chennai, asymptomatic infections (as detected by RDT) were associated with an infection in the past year. A recent study in a low endemicity area in Vietnam followed 356 persons with asymptomatic subpatent infections; 61% remained afebrile during the 24-month follow up period. They noted a median duration of asymptomatic submicroscopic *P. falciparum* infection of 2 months, with variable parasite densities; for *P. vivax* this was 6 months [28]. Like the study presented here, parasite densities among febrile participants with *P. falciparum* were higher than among non-febrile participants [28]. In a study in Thailand, 80.5% of the 41 persons with malaria detected by PCR (prevalence 3.0%) had no complaints 5 months before, during or after the survey (follow up 6–15 months with weekly fever surveillance), three (7.3%) had fever at presentation, and five (12.2%) developed fever 1–4 months after presentation [29]. A relationship between asymptomatic infections with anaemia in any of the sites was not apparent, but in Rourkela a significantly lower haemoglobin level (but not anaemia) was detected among persons with asymptomatic parasites.

Although malaria prevalence was similar in Nadiad and Rourkela, the proportion of asymptomatic infections was very low in Nadiad compared to Rourkela, and almost all malaria in Nadiad was accompanied by symptoms. It has been suggested that the asymptomatic reservoir could be minimal when transmission has decreased over many years and people have lost immunity [30] and this might be the case in Nadiad, where 5% of persons had a history of malaria in the past year. In Rourkela, 25% of the population had a history of malaria in the past year and *P. falciparum* was the main species; it was the only site with considerable bed net use. Malaria is decreasing in Rourkela [31], but the decrease in transmission may be more rapid than the loss of immunity, resulting in this significant reservoir of asymptomatic carriers; this may also be the case for Chennai [30, 32]. Immunity is affected by previous exposure to malaria, age, virulence, and number of infecting strains [16, 30, 33]. Only at one site, Rourkela, could factors affecting asymptomatic malaria be explored. As detected by microscopy and RDT, it was associated with young age, which was contrary to the expectations that older people have more immunity and will be able to better control malaria [33, 34]. Alternatively, asymptomatic infections may be present in older age groups but less likely to be detected because of lower parasite densities as a result of the higher immunity [34]. It has been suggested that asymptomatic infections may maintain the parasite during low transmission seasons [30, 34]; in Rourkela, they were more common in the rainy season (as detected by PCR). *Plasmodium vivax* infections were more likely to be asymptomatic compared to *P. falciparum* infections; results in the literature concerning this have been conflicting [33]. It is possible that the limited number of circulating parasite strains in low transmission settings induces tolerance among persons at risk of infection and re-exposure, resulting in a higher proportion of asymptomatic infections as has been suggested for Pacific Islands; a limited number of strains may be circulating in Chennai, but data are not yet available to examine this [30, 35]. Use of the term “chronic infections” instead of “asymptomatic infections” has been proposed because of the potential adverse effects on health of asymptomatic infections [15, 16]. Asymptomatic infections can be transmitted to *Anopheles* mosquitoes [33] although gametocytaemia was very low in our studies; e.g., in Rourkela, prevalence was similar in symptomatic and asymptomatic infections. It is not clear if total removal of asymptomatic malaria is needed to achieve elimination [30, 36].

Asymptomatic malaria is often defined as a positive malaria test in the absence of documented fever or a history of fever [37]. This study shows the importance of using an appropriate case-definition for asymptomatic infections: fever, chills, aches, and headaches identified all symptomatic malaria cases in Chennai, and most in Nadiad. However, symptoms cannot replace testing for the presence of *Plasmodium* parasites, and the definition of symptomatic/asymptomatic infections is mainly useful in the surveillance context for the evaluation of malaria burden. In Rourkela, there remained a group of persons who had symptoms such as cough or backache and a positive *Plasmodium* test. It is possible that these symptoms were unrelated to malaria but due to other diseases, given the higher prevalence of asymptomatic *Plasmodium* infections in this area.

## Limitations and strength

In Chennai, the number of malaria cases was low (n = 24) and in Nadiad, the number of asymptomatic malaria cases was low (n = 12); this limited the type of analyses that could be conducted. The participants with asymptomatic infections were not followed over time and so it was not possible to assess their subsequent clinical status. The study systematically asked questions concerning a series of symptoms at enrolment, not just fever, and used the same design, questionnaires, and malaria tests in all three sites, strengthening the comparability of the results.

## Conclusion

This current study reports on regional differences in general complaints at the three sites in India and on difference of malaria and asymptomatic infections by region. Asymptomatic infections are important because it can continue transmission, hinder elimination, and chronically affect people’s health. Strategies to reduce asymptomatic infections include “mass screen and treat” campaigns, mass drug administration, and reactive case management, and all these options can be combined with indoor residual spraying or ITN. Reactive case detection (RCD), the screening of persons in the households and neighborhoods of positive index cases, has been studied in Chennai and Nadiad but results were not encouraging [38]. RCD is labor intensive, is likely to miss the subpatent and submicroscopic infections depending on the malaria test and may not be effective for *P. vivax* where dormant hypnozoites in the liver can continue transmission. Mass drug treatment has been an alternative but may result in ineffective exposure of persons to adverse events of medication when the malaria prevalence is very low, and may select resistant parasites, especially when conducted in the dry season [34]. Mass screening and treatment is an alternative option; however, disadvantages are similar to RCD. A focused approach may be more effective when clusters have been identified in place and time. In Nadiad, given that the majority of malarious persons present with symptoms and there is a very low prevalence of subpatent and submicroscopic infections, prompt case-detection and effective treatment may reduce malaria cases further. In Chennai, the prevalence in the community is low but the proportion of asymptomatic persons is relatively high and additional action may be needed. In Rourkela, focusing on “hotspots” and at-risk population groups may assist in further reduction of malaria, for example the malaria camp approach currently being used by Odisha State Government to reduce malaria as part of a Durgama Anchalare Malaria Nirakaran (DAMaN) **‘**malaria control in inaccessible areas’ programme.

## Supplementary information

**Additional file 1.** Additional tables.

## Data Availability

The datasets generated and/or analysed during the current study are available in the Clinical Epidemiology Database, ClinEpiD (https://clinepidb.org), part of the EuPathDB project.

## Abbreviations

CI: Confidence interval
DAMaN: Durgama Anchalare Malaria Nirakaran
ITN: Insecticide treated net
NVBCDP: National Vector Borne Disease Control Programme
PCR: Polymerase chain reaction
RCD: Reactive case detection
RDT: Rapid diagnostic test
