# Supplementary material for: Defining symptoms of malaria in India in an era of asymptomatic infections
Source: Malar J. 2020 Jul 6;19:237. doi: 10.1186/s12936-020-03310-9 (PMC7339403; doi:10.1186/s12936-020-03310-9)
Supplement: Supplementary file 1 — Additional file 1. Additional tables. [file 12936_2020_3310_MOESM1_ESM.docx]

**Table S1A The association between age, gender and season and selected symptoms in Chennai in India, 2013-2015***

|  | **Age group** | **Risk ratio**  **(95% CI)** | **p-value** | **Gender** | **Risk ratio**  **(95% CI)** | **p-value** | **Season** | **Risk ratio**  **(95% CI)** | **p-value** |
| --- | --- | --- | --- | --- | --- | --- | --- | --- | --- |
| **Chennai** |  |  |  |  |  |  |  |  |  |
| Headache | 0-4 | 0.84, 0.24-2.95 | 0.782 |  |  |  |  |  |  |
|  | 5-14 | 1.04, 0.59-1.85 | 0.882 | Male | 0.67, 0.43-1.05 | 0.081 | Rainy | 0.76, 0.49-1.18 | 0.223 |
|  | 15+ years | Reference |  | Female | Reference |  | Dry | Reference |  |
| Chills | 0-4 |  |  |  |  |  |  |  |  |
|  | 5-14 | 1.61, 0.76-3.43 | 0.213 | Male | 0.81, 0.39-1.70 | 0.578 | Rainy | 0.66, 0.31-1.41 | 0.281 |
|  | 15+ years | Reference |  | Female | Reference |  | Dry |  |  |
| Aches | 0-4 |  |  |  |  |  |  |  |  |
|  | 5-14 | 1.18, 0.62-2.24 | 0.607 | Male | 0.81, 0.48-1.34 | 0.404 | Rainy | 0.84, 0.51-1.39 | 0.504 |
|  | 15+ years | Reference |  | Female | Reference |  | Dry |  |  |
| Cough | 0-4 | **2.98, 1.40-6.33** | 0.005 |  |  |  |  |  |  |
|  | 5-14 | **1.81, 1.02-3.20** | 0.042 | Male | 0.87, 0.56-1.34 | 0.525 | Rainy | 0.81, 0.51-1.28 | 0.365 |
|  | 15+ years | Reference |  | Female | Reference |  | Dry | Reference |  |
| Vomiting | 0-4 |  |  |  |  |  |  |  |  |
|  | 5-14 | **2.72, 1.04-7.16** | 0.042 | Male | 0.89, 0.37-2.18 | 0.804 | Rainy | 0.50, 0.20-1.27 | 0.145 |
|  | 15+ years | Reference |  | Female | Reference |  | Dry | Reference |  |
| Dizziness | 0-4 |  |  |  |  |  |  |  |  |
|  | 5-14 | 0.98, 0.15-6.29 | 0.983 | Male | 0.39, 0.09-1.69 | 0.207 | Rainy | 0.83, 0.29-2.35 | 0.721 |
|  | 15+ years | Reference |  | Female | Reference |  | Dry | Reference |  |
| Fatigue | 0-4 |  |  |  |  |  |  |  |  |
|  | 5-14 | 0.62, 0.10-3.84 | 0.609 | Male | 0.90, 0.33-2.43 | 0.833 | Rainy | 1.18, 0.45-3.10 | 0.734 |
|  | 15+ years | Reference |  | Female | Reference |  | Dry | Reference |  |
| Fever in the past 48 hrs | 0-4 | 1.24, 0.44-3.49 | 0.684 |  |  |  |  |  |  |
|  | 5-14 | 1.39, 0.81-2.39 | 0.226 | Male | 0.72, 0.46-1.14 | 0.158 | Rainy | 0.69, 0.43-1.10 | 0.121 |
|  | 15+ years | Reference |  | Female | Reference |  | Dry | Reference |  |
| Documented fever | 0-4 | 1.21, 0.43-3.41 | 0.715 |  |  |  |  |  |  |
|  | 5-14 | 1.55, 0.94-2.57 | 0.088 | Male | 0.76, 0.49-1.18 | 0.220 | Rainy | 0.64, 0.41-1.01 | 0.057 |
|  | 15+ years | Reference |  | Female | Reference |  | Dry | Reference |  |

**Table S1B The association between age, gender and season and selected symptoms in Nadiad in India, 2013-2015***

|  | **Age group** | **Risk ratio**  **(95% CI)** | **p-value** | **Gender** | **Risk ratio**  **(95% CI)** | **p-value** | **Season** | **Risk ratio**  **(95% CI)** | **p-value** |
| --- | --- | --- | --- | --- | --- | --- | --- | --- | --- |
| Headache | 0-4 | 0.61, 0.25-1.52 | 0.288 |  |  |  |  |  |  |
|  | 5-14 | 1.02, 0.78-1.32 | 0.895 | Male | 1.02, 0.84-1.23 | 0.850 | Rainy | **0.49, 0.40-0.61** | <0.001 |
|  | 15+ years | Reference |  | Female | Reference |  | Dry | Reference |  |
| Chills | 0-4 | **1.80, 1.06-3.07** | 0.029 |  |  |  |  |  |  |
|  | 5-14 | **1.42, 1.09-1.86** | 0.011 | Male | 1.22, 0.97-1.54 | 0.096 | Rainy | **0.68, 0.54-0.87** | 0.002 |
|  | 15+ years | Reference |  | Female | Reference |  | Dry | Reference |  |
| Aches | 0-4 |  |  |  |  |  |  |  |  |
|  | 5-14 | 1.02, 0.68-1.53 | 0.920 | Male | 1.02, 0.76-1.36 | 0.894 | Rainy | **0.44, 0.32-0.61** | <0.001 |
|  | 15+ years | Reference |  | Female | Reference |  | Dry | Reference |  |
| Cough | 0-4 | NA |  |  |  |  |  |  |  |
|  | 5-14 | **3.60, 2.10-6.18** | <0.001 | Male | 1.41, 0.83-2.38 | 0.200 | Rainy | **0.39, 0.22-0.69** | <0.001 |
|  | 15+ years | Reference |  | Female | Reference |  | Dry | Reference |  |
| Vomiting | 0-4 |  |  |  |  |  |  |  |  |
|  | 5-14 | 1.33, 0.69-2.59 | 0.395 | Male | 1.28, 0.74-2.20 | 0.381 | Rainy | 1.01, 0.59-1.73 | 0.971 |
|  | 15+ years | Reference |  | Female | Reference |  | Dry | Reference |  |
| Dizziness | 0-4 | NA |  |  |  |  |  |  |  |
|  | 5-14 | **2.36, 1.11-4.98** | 0.025 | Male | 0.71, 0.35-1.46 | 0.349 | Rainy | 1.43, 0.71-2.87 | 0.320 |
|  | 15+ years | Reference |  | Female | Reference |  | Dry | Reference |  |
| Fatigue | 0-4 |  |  |  |  |  |  |  |  |
|  | 5-14 | 1.17, 0.54-2.56 | 0.690 | Male | 1.06, 0.60-1.88 | 0.848 | Rainy | 1.44, 0.79-2.64 | 0.234 |
|  | 15+ years | Reference |  | Female | Reference |  | Dry | Reference |  |
| Fever in past 48 hours | 0-4 | 1.31, 0.61-2.85 | 0.488 |  |  |  |  |  |  |
|  | 5-14 | **1.44, 1.05-1.97** | 0.023 | Male | 1.14, 0.87-1.50 | 0.329 | Rainy | 0.91, 0.70-1.19 | 0.490 |
|  | 15+ years | Reference |  | Female | Reference |  | Dry | Reference |  |
| Documented fever | 0-4 | 0.74, 0.11-5.01 | 0.754 |  |  |  |  |  |  |
|  | 5-14 | **2.47, 1.38-4.44** | 0.002 | Male | 1.63, 0.94-2.84 | 0.083 | Rainy | 0.90, 0.51-1.56 | 0.694 |
|  | 15+ years | Reference |  | Female | Reference |  | Dry | Reference |  |

**Table S1C The association between age, gender and season and selected symptoms in Rourkela in India, 2013-2015***

| **Rourkela** | **Age group (years)** | **Risk ratio**  **(95% CI)** | **p-value** | **Gender** | **Risk ratio**  **(95% CI)** | **p-value** | **Season** | **Risk ratio**  **(95% CI)** | **p-value** |
| --- | --- | --- | --- | --- | --- | --- | --- | --- | --- |
| Headache | 0-4 |  |  |  |  |  |  |  |  |
|  | 5-14 | 0.54, 0.26-1.11 | 0.093 | Male | **0.71, 0.52-0.96** | 0.027 | Rainy | **3.35, 2.42-4.65** | <0.001 |
|  | 15+ years | Reference |  | Female | Reference |  | Dry | Reference |  |
| Chills | 0-4 | **3.75, 2.33-6.04** | <0.001 |  |  |  |  |  |  |
|  | 5-14 | **2.66, 1.71-4.14** | <0.001 | Male | **1.54, 1.05-2.28** | 0.029 | Rainy | **2.12, 1.44-3.14** | <0.001 |
|  | 15+ years | Reference |  | Female | Reference |  | Dry | Reference |  |
| Aches | 0-4 | **0.12, 0.03-0.42** | 0.001 |  |  |  |  |  |  |
|  | 5-14 | **0.50, 0.32-0.77** | 0.002 | Male | **0.55, 0.41-0.74** | <0.001 | Rainy | **1.83, 1.33-2.50** | <0.001 |
|  | 15+ years | Reference |  | Female | Reference |  | Dry | Reference |  |
| Cough | 0-4 | **4.21, 2.73-6.49** | <0.001 |  |  |  |  |  |  |
|  | 5-14 | **1.61, 1.01-2.57** | 0.045 | Male | 1.41, 0.97-2.05 | 0.076 | Rainy | **1.85, 1.27-2.69** | 0.001 |
|  | 15+ years | Reference |  | Female | Reference |  | Dry | Reference |  |
| Vomiting | 0-4 | 0.69, 0.09-5.46 | 0.726 |  |  |  |  |  |  |
|  | 5-14 | 3.33, 0.95-11.68 | 0.060 | Male | 0.75, 0.26-2.17 | 0.601 | Rainy | 0.70, 0.23-1.98 | 0.480 |
|  | 15+ years | Reference |  | Female | Reference |  | Dry | Reference |  |
| Dizziness | 0-4 |  |  |  |  |  |  |  |  |
|  | 5-14 | 0.47, 0.08-2.89 | 0.417 | Male | 1.56, 0.66-3.68 | 0.309 | Rainy | **7.50, 2.41-23.32** | <0.001 |
|  | 15+ years | Reference |  | Female | Reference |  | Dry | Reference |  |
| Fatigue | 0-4 |  |  |  |  |  |  |  |  |
|  | 5-14 | **0.15, 0.05-0.43** | <0.001 | Male | 0.68, 0.45-1.02 | 0.063 | Rainy | **1.58, 1.07-2.33** | 0.020 |
|  | 15+ years | Reference |  | Female | Reference |  | Dry | Reference |  |
| Fever in past 48 hours | 0-4 | **1.80, 1.05-3.10** | 0.033 |  |  |  |  |  |  |
|  | 5-14 | **2.19, 1.50-3.20** | <0.001 | Male | 1.00, 0.71-1.40 | 0.985 | Rainy | **2.27, 1.59-3.25** | <0.001 |
|  | 15+ years | Reference |  | Female | Reference |  | Dry | Reference |  |
| Documented fever | 0-4 | **3.47, 2.02-5.97** | <0.001 |  |  |  |  |  |  |
|  | 5-14 | **5.61, 3.85-8.19** | <0.001 | Male | 0.78, 0.56-1.09 | 0.149 | Rainy | 1.34, 0.96-1.87 | 0.088 |
|  | 15+ years | Reference |  | Female | Reference |  | Dry | Reference |  |

CI: confidence interval; NA: not applicable (0-value); NS: not significant

*Multivariate weighted models adjusted for age, gender and season. Documented fever: measured axillary body temperature at enrolment and documented fever defined as a body temperature of ≥37 °C). If for the age group 0-4 years the risk ratio is missing, this means there were empty cells in the table (value of 0 for the complaint) and the risk ratio could not be assessed. In bold the estimates and confidence intervals where 1 (no difference) is not included.
